# Met expression is an independent prognostic risk factor in patients with oesophageal adenocarcinoma

**DOI:** 10.1038/sj.bjc.6604251

**Published:** 2008-03-18

**Authors:** J B Tuynman, S M Lagarde, F J W ten Kate, D J Richel, J J B van Lanschot

**Affiliations:** 1 Department of Surgery, Academic Medical Centre at the University of Amsterdam, Amsterdam, The Netherlands; 2 Department of Medical Oncology, Academic Medical Centre at the University of Amsterdam, Amsterdam, The Netherlands; 3 Department of Pathology, Academic Medical Centre at the University of Amsterdam, Amsterdam, The Netherlands; 4 Department of Surgery, Erasmus Medical Centre, Rotterdam, The Netherlands

**Keywords:** oesophageal adenocarcinoma, Met, HGF receptor, COX-2, prognosis

## Abstract

Oesophageal adenocarcinoma is an aggressive malignancy with propensity for early lymphatic and haematogenous dissemination. Since conventional TNM staging does not provide accurate prognostic information, novel molecular prognostic markers and potential therapeutic targets are subject of intense research. The aim of the present study was to study the prognostic significance of Met, the hepatic growth factor (HGF) receptor and a possible target for therapy in comparison to cyclooxygenase-2 (COX-2). Tumour sections from 145 consecutive patients undergoing intentionally curative surgery for oesophageal adenocarcinoma were immunohistochemically analysed for Met and COX-2 expression. Clinicopathological data were prospectively collected for all patients. Patients with high Met expression had significantly reduced overall and disease-specific 5-year survival rates (*P*⩽0.001 and *P*⩽0.001, respectively) and were more likely to develop distant metastases (*P*=0.002) and local recurrences (*P*=0.004) compared to patients with low Met expression. High COX-2 expression tended to be correlated with poor long-term survival but this did not reach statistical significance. Expression of Met was recognised as a significant and independent prognostic factor by stage-specific analysis and multivariate analysis (relative risk=2.3; 95% CI=1.3–4.1). These findings support the importance of Met in oesophageal adenocarcinoma and support the concept of Met tyrosine kinase inhibition as (neo-) adjuvant treatment.

Oesophageal adenocarcinoma (OA) is a highly aggressive malignancy with early lymphatic and haematogenous dissemination. The incidence of OA is increasing rapidly in the Western World (Enzinger and Mayer, 2003). Despite advances in diagnosis and treatment of the disease, even after potentially curative surgery the overall 5-year survival rate rarely exceeds 35% (Hulscher *et al*, 2002; Cunningham *et al*, 2006). Subgroup analysis in early-stage tumours have shown good survival rates although even early-stage tumours show early lymphatic dissemination.

For adenocarcinoma of the oesophagus the most important conventional prognostic factors are summarised in the pTNM stage of the oesophagus. Also other pathological aspects such as extracapsular lymph node involvement and (relative) number of positive nodes have prognostic impact. However, these conventional prognostic factors have limited accuracy (Lagarde *et al*, 2006). Therefore, molecular prognostic markers, which can serve as targets for therapy are subject of intense research. For OA only few molecular prognostic factors have been identified and molecular events responsible for the development of lymphatic and haematogenous dissemination are still poorly understood (Lagarde *et al*, 2006). Identification of growth factor receptors with tyrosine kinase activity, highly expressed in advanced cancer, has been shown to provide both prognostic information and potential molecular targets for (neo-) adjuvant therapy (Krause and Van Etten, 2005). A promising development in cancer therapy is the combination of surgery with potent selective growth factor receptor inhibitors as (neo-) adjuvant therapy resulting in improved overall and disease-specific survival (Verweij *et al*, 2004; Krause and Van Etten, 2005; Gold and Dematteo, 2006; Motzer *et al*, 2007; Smith *et al*, 2007). Therapeutic usage of small molecules selectively inhibiting c-KIT, a growth factor receptor present in gastrointestinal stromal cell tumours (GIST), has resulted in remarkable responses and has enhanced prognosis for patients with GIST to a great extent (Gold and Dematteo, 2006). Other examples of targeted (neo-) adjuvant therapy are the inhibition of vascular endothelial growth factor receptor (VEGFr) in patients with advanced colorectal cancer, the inhibition of HER2-Neu, an epidermal growth factor receptor in patients with breast cancer and the inhibition of both VEGFr and platelet-derived growth factor receptor (PDGFr) in patients with renal cell carcinoma (Hurwitz *et al*, 2004; Verweij *et al*, 2004; Geyer *et al*, 2006; Motzer *et al*, 2007).

Growth factor receptors have been identified in OA and some show higher expression in later stages of cancer development (Lagarde *et al*, 2006; Vallbohmer *et al*, 2006). However, the prognostic significance of growth factors expressed in OA has only been investigated in relatively small patient cohorts and no significance in multivariate analysis was demonstrated so far. The only independent molecular prognostic factor demonstrated for OA is cyclooxygenase-2 (COX-2) expression as published by our group (Buskens *et al*, 2002; Lagarde *et al*, 2006). Recently, we have reported a clinical study in which neo-adjuvant selective COX-2 inhibition downregulates Met expression in conjunction with COX-2 expression in patients with OA (Tuynman *et al*, 2005). Met is the hepatocyte growth factor (HGF) receptor and is identified in OA (Tuynman *et al*, 2005; Anderson *et al*, 2006). Overexpression of Met and/or its ligands has been shown to contribute to progression and dissemination of several malignancies including lung, colorectal, gastric, breast, prostate, thyroid, pancreas, and oesophageal cancer (Hu *et al*, 2001; Saeki *et al*, 2002; Christensen *et al*, 2003; Murai *et al*, 2004; Anderson *et al*, 2006). In experimental models, activation of Met (endogenously by mutations in its tyrosine kinase domain, or exogenously by HGF and prostaglandins produced by COX-2) causes decreased apoptosis and enhanced proliferation, angiogenesis, and invasion (Shinomiya *et al*, 2004; Herrera *et al*, 2005; Boccaccio and Comoglio, 2006). Thus, COX-2 and Met seem functionally connected. In cancer development, COX-2 is present in early stages of dysplasia, initiating cancer growth and progression whereas Met is an important key regulator of molecular processes in later stages of cancer development and progression (Tuynman *et al*, 2004). Small molecules selectively inhibiting Met have been shown to inhibit dissemination and cancer growth both *in vitro* as in animal studies (Christensen *et al*, 2003; Kim *et al*, 2003; Hov *et al*, 2004; Herrera *et al*, 2005; Martens *et al*, 2006; Watson *et al*, 2006). Consequently, inhibition of Met as (neo-) adjuvant therapy for OA seems a promising strategy.

A relation between Met expression and stage of disease has been described previously (Anderson *et al*, 2006). However, the potential value of Met expression in OA as an independent prognosticator calculated by multivariate analysis has not yet been addressed in a large consecutive cohort. Therefore, the aim of the present study was to characterize further the prognostic significance of Met expression in a large consecutive cohort of patients with OA.

## PATIENTS AND METHODS

### Patients

A consecutive series of 306 patients who underwent potentially curative oesophagectomy at the Department of Surgery of the Academic Medical Centre at the University of Amsterdam, The Netherlands for adenocarcinoma of the mid/distal oesophagus between January 1993 and December 2000 was selected. Preoperative workup included endoscopy with histological biopsy, external ultrasonography of the abdomen and neck, CT scan of the abdomen and chest, radiography of the chest, oesophageal endosonography, and indirect laryngoscopy. Lymph node metastases at the coeliac trunk were a contraindication for resection only when considered non-resectable (i.e., larger than 2 cm in diameter) and confirmed by cytological puncture. Patients did not receive additional (neo-) adjuvant chemotherapy and/or radiotherapy. Clinicopathological data from all operated patients were permanently prospectively collected. Follow-up was complete for all patients and extended until July 2006, ensuring a minimal potential follow-up of 5.5 years. Recurrence of disease was diagnosed on clinical grounds. However, whenever a relapse was suspected, radiologic, endoscopic, or histological confirmation was sought. Recurrent disease was classified as locoregional (occurring in the upper abdomen or mediastinum) or distant (including cervical recurrences). All pathology reports were reviewed to identify those patients in whom the adenocarcinoma had developed in a histologically proven Barrett's segment (defined by the presence of goblet cells). Patients with an adenocarcinoma of the cardia or gastro-oesophageal junction without a clear Barrett's segment were excluded (*n*=161). This careful selection of patients has been described in our previous report (Buskens *et al*, 2002). Archival materials of the remaining 145 patients were re-evaluated to obtain the sample with deepest invasion of each tumour.

### Surgical tissue specimens

All 145 patients were treated with subtotal oesophagectomy and resection of the lesser curvature of the stomach. Between April 1994 and February 2000, 96 patients (66%) were randomly assigned to either transhiatal or transthoracic oesophagectomy as part of a randomized trial comparing both techniques (Hulscher *et al*, 2002). In the remaining 49 patients, a standard transhiatal procedure was performed. In 95 patients (65.5%), resection was performed by a transhiatal approach without thoracotomy. Lymphadenectomy comprised *en bloc* removal of all lymphatic tissue in the lower posterior mediastinum, along the cardia and the lesser curvature of the stomach. Fifty patients (34.5%) underwent oesophagectomy through a right-sided thoracotomy followed by a laparotomy in combination with two-field lymph node dissection. This procedure included an abdominal lymphadenectomy as described plus the removal of lymph nodes along the common hepatic artery, the splenic artery, and the coeliac trunk as well as an extended lymph node dissection in the chest (i.e., including the right paratracheal, infra-aortic arch, and subcarinal lymph nodes).

### Immunohistochemistry

Of all patients 5-*μ*m thick sections of paraffin and formaldehyde-fixed tissue of the resection specimens were cut. For immunohistochemical staining, sections were incubated overnight at 37°C and subsequently deparaffinised in xylene, rehydrated, and treated with 3% H_2_O_2_ in methanol for 10 min to block endogenous peroxidase activity. All specimens were subjected to heat-induced antigen retrieval in 10 mM sodium citrate buffer (pH 6.0) for 10 min at 95°C. To block aspecific binding the slides were incubated with Tris-buffered saline (TBS) supplemented with 5% goat serum. Sections were incubated with the primary antibodies anti-Met c-Met (3D4; Zymed, San Francisco, CA, USA) (1 : 100), and anti-human COX-2 (160112; Cayman Chemical Co., Ann Arbor, MI, USA) (1 : 200) diluted in TBS with 1% bovine serum albumin overnight at 4°C. For the Met staining the sections were incubated after washing steps with anti-mouse/rabbit-peroxidase polymer for 30 min at room temperature (Powervision; Immunovision Inc., Daly City, CA, USA). Diaminobenzidine chromogen (Sigma, St Louis, MO, USA) was used for visualisation. For the COX-2 staining the sections were treated with biotinylated horse anti-mouse immunoglobulin (1 : 200; Vector Laboratories Inc., Burlingame, CA, USA) and avidin–biotin peroxidase complex (Vectastain ABComplex; Vector Laboratories). After these steps for Met and COX-2 staining the sections were counterstained with haematoxylin and embedded. Specificity of the antibodies was confirmed by controls using irrelevant immunoglobulins instead of primary antibodies. Colon cancer tissue was included as a positive control.

### Scoring

**Figure 1:**
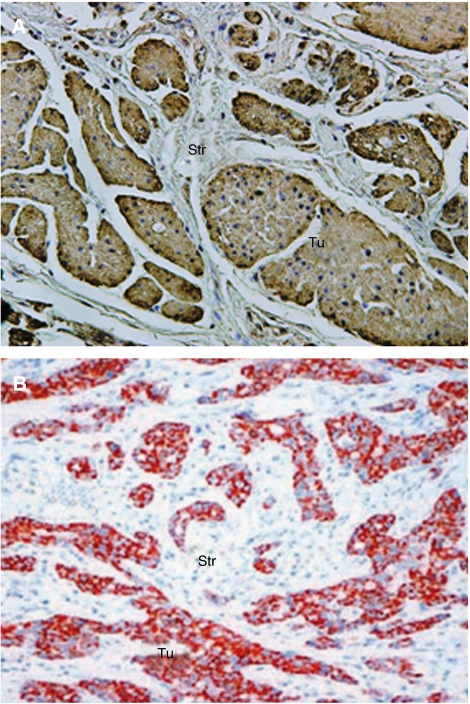
Representive samples of immunohistochemical staining of Met (**A**) and COX-2 (**B**) (Str=stroma, T=tumour).

Met and COX-2 immunohistochemical staining were scored semiquantitatively using a four-step scale as used and validated in previous reports (Buskens *et al*, 2002; Tuynman *et al*, 2005). The following scoring criteria of tumour cells were agreed upon before the analysis: (0) no staining or equal to background; (1) weak diffuse cytoplasmic staining (may contain stronger intensity in less than 10% of cancer cells); (2) moderate granular cytoplasmic staining in 10–90% of cancer cells; (3) over 90% of tumour cells stained with strong intensity (Figure 1).

The analysis of all tissue sections was performed independently by three different investigators (JBT, SML, and FJWTK) without patient identification parameters to correct for observer accuracy. The semiquantitative scoring by the investigators had a low observer variation; 92% of the specimens were categorized identically. In cases of disagreement (*n*=6 for COX-2 expression and *N*=13 for Met expression) consensus was reached after re-evaluation by the investigators using a multiheaded microscope. Data regarding COX-2 staining intensity are equal as previously described and used for the present analysis. Areas of diffuse haemorrhage or necrosis were neglected.

### Statistics

Statistical calculations were performed using SPSS version 14.0 (Statistical Package for the Social Sciences, Chicago, IL, USA). The association between demographic and clinicopathological features and protein expression was analysed using Student's *t*-test (continuous data) and *χ*^2^-test (categorical data). Overall and disease-specific 5-year survival rates were estimated according to the Kaplan–Meier method and compared between groups using the log-rank test. Overall survival was calculated using deaths since time of surgery irrespective of cause. For disease-specific survival all non-disease-related deaths were excluded including in-hospital death within 90 days of surgery, since we assumed that these patients had died because of comorbidity and surgery-related causes. The Cox proportional hazards regression model was used to identify prognostic factors. To identify independent prognostic factors multivariate Cox regression analysis was carried out. Variables with multiple categories were recoded into dichotomous variables by combining categories with a comparable prognosis (differentiation grade, good *vs* moderate and poor (poor); tumour T stage, stage 1 and 2 *vs* 3; Met expression, no or weak staining (low) *vs* moderate to strong staining (high); COX-2 expression, no or weak staining (low) *vs* moderate to strong staining (high).

## RESULTS

A total of 145 consecutive patients with OA were included for immunohistochemical analysis. Of these patients 120 were men (83%) and 25 were women (17%) with a median age of 67 years (range=35–85) (Table 1). The majority of patients (*N*=83, 57%) had a T3 tumour and 80 patients (55%) had positive lymph nodes. The overall 5-year survival in the included group was 35% and the disease-specific 5-year survival was 48%. Two patients (1.4%) died within 90 days due to post-operative complications (myocardial and respiratory failure in one patient and cerebrovascular event in another one patient).

High Met staining (as opposed to low Met staining) was observed in 78 cases (54%). Of these 78 patients, 28 cases were scored as strong Met expression and 50 as moderate Met expression. In 67 patients (46%) Met expression was classified as low; 56 patients had weak Met expression and 11 patients had no or equal to background staining of Met. Met expression was mainly localised in neoplastic cells (Figure 1A and B) but was also weakly identified in non-neoplastic epithelial cells (both squamous and columnar epithelium) and in stromal cells. Interobserver variation was 8% for Met expression. All specimens that were discrepant (*n*=13) were re-evaluated and the consensus score was used for further analysis. Results of COX-2 expression have been described previously in this cohort of patients. Briefly, COX-2 expression was negative to weak in 21% (COX-2 low) and moderate to strong in 79% (COX-2 high) of the carcinomas (Buskens *et al*, 2002; Tuynman *et al*, 2005).

High Met expression was observed more often in patients with higher T stage (*P*=0.003), in patients with positive lymph nodes (*P*⩽0.001) and a poor differentiation grade (*P*=0.003) (Table 1). Met expression was not correlated with COX-2 expression (*P*=0.839).

During 5-year follow-up, 92 patients died: 17 patients died of unrelated causes and 75 patients died of recurrent disease. Of these patients, 23 had locoregional recurrences, 39 patients had haematogenous recurrences and 13 patients had both locoregional and haematogenous recurrences.

**Figure 2:**
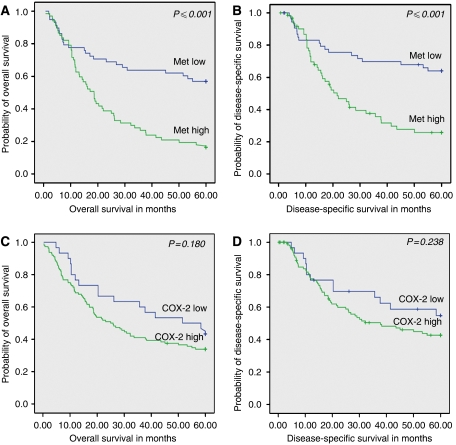
Kaplan–Meier survival curves of 145 patients with adenocarcinoma of the oesophagus. Patients with high Met expression had a significantly worse overall 5-year survival. (**A**) (*P*⩽0.001) and disease-specific 5-year survival (*P*⩽0.001) (**B**) as compared to patients with low Met expression. Overall 5-year survival and disease-specific 5-year survival tended to be worse in patients with high COX-2 expression (**C** and **D**, respectively) as compared to patients with low COX-2 expression but this did not reach statistical significance (*P*=0.180 and *P*=0.238, respectively).

After a complete follow-up, overall 5-year survival was significantly lower in patients with high Met expression as compared to patients with low Met expression; 16 *vs* 57% (*P*⩽0.001). Furthermore, disease specific 5-year survival was significantly lower in patients with high Met expression as compared to patients with low Met expression; 33 *vs* 66% (*P*⩽0.001). Patients with high Met expression were more likely to develop distant metastases (*P*=0.002) as well as local recurrences (*P*=0.004). Patients with high COX-2 expression tended to have a poor overall and disease-specific 5-year survival as compared to patients with low COX-2 expression but in contrast to previous reports this did not reach statistical significance (Figure 2). Univariate analysis revealed that T stage, N stage, M1a stage, differentiation grade, and Met expression were all significant prognostic indicators for disease-specific 5-year survival (Table 2). Multivariate analysis of these variables demonstrated that T3 stage (relative risk (RR)=1.9, (95% confidence interval (95% CI=1.0–3.5)), (*P*=0.035)), lymph node involvement (RR=2.8, (95% CI=1.5–5.3), (*P*=0.001)) and high Met expression (RR=2.3, (95% CI=1.3–4.1), (*P*=0.004)) were independent prognostic factors (Table 3).

**Figure 3:**
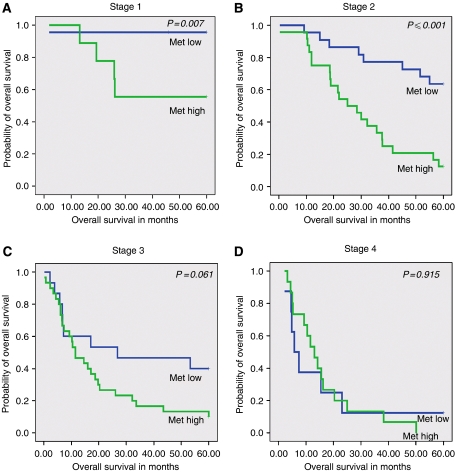
Stage specific Kaplan–Meier survival curves for high *vs* low Met expression. Patients with stage 1 (T1, N0, and M0) (**A**) and stage 2 (T2, 3, N0, M0 or T1, N1, M0) (**B**) and high Met expression had a significantly worse overall 5-year survival as compared to patients with stage 1 or 2 with low Met expression (*P*=0.007 and *P*⩽0.001, respectively). Patients with stage 3 (T3, N1, M0 or T4, N0, 1, M1a) (**C**) with high Met expression had a worse overall 5-year survival as compared to patients with stage 3 with low Met expression however, this did not reach statistical significance (*P*=0.061). High or low Met expression in patients with stage 4 OA did not change the 5-year overall survival (*P*=0.915) (**D**).

Subgroup analysis in patients with Stages 1 and 2 OA revealed that overall 5-year survival was significantly lower in patients with high Met expression as compared to patients with low Met expression (*P*=0.007 and *P*⩽0.001, respectively) (Figure 3). Patients with stage 3 disease and high Met expression tended to have poor overall 5-year survival as compared to patients with low Met expression but no statistical significance was reached (*P*=0.064). In contrast to patients with stage 1 and 2, in patients with stage 4 OA the Met expression level did not discriminate poor *vs* better overall 5-year survival.

## DISCUSSION

This study provides evidence that Met expression level (as detected by immunohistochemical analysis) is an independent prognostic factor in OA. Overall 5-year survival after potentially curative resection is significantly worse in patients with tumours expressing high Met levels compared to low Met levels.

In literature, lymphatic dissemination as identified on histopathological examination is the single most important prognostic factor in patients with oesophageal cancer (Lagarde *et al*, 2006). Also in the present study, lymph node involvement is a strong independent prognostic factor next to T stage and Met expression level. Since Met expression was correlated stage of disease the subgroup analysis revealed that especially in stage 1 and 2 OA Met expression is a significant and valuable prognostic factor. In stage 4 disease, Met expression level did not discriminate poor *vs* good 5-year overall survival indicating that in advanced stage OA other factors determine survival. Since Met expression appears to be an important independent prognosticator and especially, this might offer an attractive opportunity for targeted therapy. Selective inhibitors of Met have recently become available and successful inhibition of tumour progression, stromal and endothelial adhesion and dissemination has been reported both *in vitro* and in animal studies. Targeted therapy of growth factor receptors has been shown clinically effective in other cancer types such as chronic myelogenous leukaemia, gastrointestinal stromal tumours, HER-2/NEU overexpressing breast cancer, colorectal cancer and non-small cell lung cancer (Verweij *et al*, 2004; Krause and Van Etten, 2005; Gold and Dematteo, 2006; Motzer *et al*, 2007; Smith *et al*, 2007).

A possible limitation of the present study is the semiquantitative evaluation of immunohistochemistry. The rational to semiquantitatively score Met and COX-2 immunohistochemically was to compare results from earlier reports. These scoring methods have been used and validated in our previous reports (Buskens *et al*, 2002; Tuynman *et al*, 2005). A significant advantage of immunohistochemistry is the cellular morphology, which helps to correct for false-positive staining (blood vessels, stromal expression etc.). Future studies using microarray gene expression technique can help to validate results obtained in this patient cohort.

Surprisingly, COX-2 expression was not a significant prognostic factor in this study. The same cohort of patients was employed for the current analysis of Met expression as reported on earlier for COX-2 expression (Buskens *et al*, 2002). In this study, a minimal follow-up of 60 months was available whereas in the previous study the median follow-up was only 27 months. Although survival in patients with high COX-2 expression tended to be poorer than that in patients with low COX-2 expression and this did not reach statistical significance. Theoretically, the difference between COX-2 expression and Met expression as prognostic indicators can probably be explained by their function. The COX-2 enzyme is enhanced in inflammation and has been shown to be involved in early progression of oesophageal metaplasia and dysplasia into (adeno-) carcinoma (Morris *et al*, 2001; Buskens *et al*, 2002; Abdalla *et al*, 2004; Ling *et al*, 2007). Increased COX-2 expression causes activation of several cancer-related genes including the HGF receptor Met (Boon *et al*, 2004; Han *et al*, 2006). Vice versa COX-2 inhibition causes downregulation of cancer-related genes including Met as it has been published previously by our group (Tuynman *et al*, 2005). In comparison to COX-2, Met is involved later in the process of cancer development and has been shown vital in cancer progression (Boccaccio and Comoglio, 2006). The proto-oncogene Met, also known as the scatter factor, has been shown particularly important in morphogenic differentiation and organisation of three-dimensional tubular structures as well as in cell growth and loss of cellular adhesion causing migration (dissemination) of cells (Boccaccio and Comoglio, 2006). Since OA is known for its propensity to early lymphatic and haematogenous dissemination, the strong prognostic significance of high Met expression for both overall and disease-specific 5-year survival can explain this clinical behaviour at least partly. These results suggest that employment of new therapeutic agents targeting Met might be of value as (neo-) adjuvant therapy in patients with OA, especially if Met expression is high.

In conclusion, our data indicate that high Met expression is a significant independent indicator of poor long-term survival in patients after potentially curative resection of OA. Targeting this receptor by a selective Met kinase inhibitor is an attractive (neo-) adjuvant treatment option that should be tested especially in patients with high tumoral Met expression.

## Figures and Tables

**Table 1:** Correlation of clinicopathological findings and Met expression

**Patient characteristics (*n*=145)**	**Overall**	**Low Met expression (*N*=67)**	**High Met expression (*N*=78)**	***P*-value**
Median age (range)	67 (35–85)	67 (35–83)	68 (44–85)	0.493
				
*Sex*				
Male (%)	120 (83%)	63 (94%)	57 (73%)	0.435
				
*Tumour characteristics*				
*T stage*^a^				0.017
T1	44 (30%)	29 (43%)	15 (19%)	
T2	18 (12%)	8 (12%)	10 (13%)	
T3	83 (57%)	30 (45%)	53 (68%)	
*N stage*^b^				⩽0.001
N0	65 (45%)	38 (57%)	27 (35%)	
N1	80 (55%)	29 (43%)	51 (65%)	
*M stage*^c^				0.086
M0	122 (84%)	58 (87%)	64 (82%)	
M1a	23 (16%)	9 (13%)	14 (18%)	
				
*Differentiation grade*				0.078
Good	11 (8%)	5 (7%)	6 (8%)	
Moderate	56 (39%)	35 (52%)	21 (27%)	
Poor	78 (54%)	27 (40%)	51 (65%)	
				
Overall 5-year survival	35%	57%	16%	⩽0.001
Disease-specific 5-year survival	48%	66%	33%	⩽0.001

a T1, tumour limited to (sub)mucosa; T2, tumour infiltrates muscularis propia; T3, tumour infiltrates adventitia layer; as determined in the pathological resection specimens.

b N0, no tumour positive locoregional lymph nodes; N1, locoregional lymph node metastasis.

c M0, no distant metastasis, M1a, metastasis in coeliac lymph nodes.

**Table 2:** Results of univariate analysis of clinical, pathological and immunohistochemical parameters related to disease-specific 5-year survival

	**Odds ratio (confidence interval)**	***P*-value**
Patient sex (male *vs* female)	1.2 (0.7–2.3)	0.435
		
Patient ASA classification (0 and 1 *vs* 2 or 3)	1.0 (0.5–2.0)	0.974
		
Patient age (70 and higher *vs* lower than 70 years)	1.1 (0.5–2.2)	0.283
		
Tumour T stage (3 *vs* 1 and 2)	4.1 (2.4–7.2)	0.001
		
Tumour N stage (1 *vs* 0)	4.9 (2.8–8.6)	0.000
tumour M1a stage (1 *vs* 0)	3.7 (2.2–6.5)	0.035
		
Differentiation grade (moderate and poor *vs* good)	2.2 (1.3–3.6)	0.015
		
Met expression (high *vs* low)	3.5 (2.0–5.9)	0.000
		
COX-2 expression (high *vs* low)	1.4 (0.8–2.6)	0.234

**Table 3:** Results of multivariate analysis of pathological and immunohistochemical parameters related to disease-specific 5-year survival according to the Cox regression model

	**Relative risk (confidence interval)**	***P*-value**
T stage (3 *vs* 1 and 2)	1.9 (1.0–3.5)	0.035
		
N stage (1 *vs* 0)	2.8 (1.5–5.3)	⩽0.001
		
M1a stage (1a *vs* 0)	1.8 (0.9–3.4)	0.056
		
Tumour differentiation grade (moderate and poor *vs* good)	1.6 (0.9–2.7)	0.077
		
Met expression (high *vs* low)	2.3 (1.3–4.1)	0.004
